# A Three-Stage Solidification Model for Food Particles

**DOI:** 10.3390/foods11010046

**Published:** 2021-12-24

**Authors:** Seshasai Srinivasan

**Affiliations:** W Booth School of Engineering Practice and Technology, McMaster University, Hamilton, ON L8S 4L8, Canada; ssriniv@mcmaster.ca

**Keywords:** cocoa butter powder, particle solidification model, heat transfer, multiphase flows, numerical analysis, experimental validation, parameter analysis

## Abstract

A three-stage solidification model for food droplets has been implemented in a computational fluid dynamics code. It comprises of an initial cooling stage that is based on the principles of convective heat transfer. This is followed by the solidification period that is initiated once the droplet cools to a phase change temperature. Finally, when the droplet is completely solidified, the tempering phase begins where the droplet cools to the temperature of the ambient air. The model has been validated with respect to the experimental data for cocoa butter. Additional simulations were made in which the crystallization behavior of the cocoa butter droplets in relation to the droplet size, ambient air temperature and the relative drop-gas velocity was investigated. It was found that the crystallization time is exponentially related to the droplet size. Further, it increased with the ambient temperature, but decreased with the relative drop-gas velocity. Overall, the results suggest operating at the extreme values of the process parameters, requiring high amount of energy, to minimize the crystallization time. It was concluded that there is a need for optimizing the operating conditions of the powder production process to minimize the energy requirement of the system while maintaining a reasonable crystallization time.

## 1. Introduction

Solidification is a multi-stage process, which can be divided into three distinct periods, viz., a pre-cooling period, a phase change period and a tempering period. In the *pre-cooling period*, the material is cooled from its initial temperature $\left(T_{i}\right)$ to a phase change temperature $\left(T_{f}\right)$. At this temperature, the *phase change period* is initiated in which all the latent heat is released. At the end of this stage, the solidified material enters a *tempering period* where the material temperature decreases further to the ambient temperature $\left(T_{a}\right)$. Theoretical approaches in studying the solidification problem include the simple Plank-type models, as well as the more advanced numerical models based on Fourier’s heat conduction equation.

The Plank’s model [1] is basically an algebraic equation to predict the solidification time. Thus, it is incapable of capturing the crystallization characteristics such as the internal temperature distribution of the material. One of the major drawbacks of this model is that it does not take into account the heat to be removed during the pre-cooling and the tempering periods. In other words, the model assumes that the material is at the phase change temperature, $T_{f}$, when it is applied. It is now generally accepted that this model under-predicts the freezing time. Several researchers have proposed numerous variations of this model to overcome this drawback. Essentially, all of them focus on modifying the latent heat term to take into account the heat rejected during the pre-cooling and the tempering stages. A detailed review of the Plank’s model and its variants for several food products is presented by Ramaswamy and Tung [2]. Since our interest is in a more sophisticated numerical model, the Plank’s approximation is not pursued further in this study.

In order to investigate the intricate details of the crystallization process a more advanced numerical approach based on Fourier’s heat conduction principles is essential. This will aid in understanding the phase change characteristics such as the propagation of the solidification front and the internal temperature distribution in the material. Further, coupling this numerical approach with crystallization models, physical processes such as dendrite growth can also be studied. Thus, using detailed numerical modeling one can obtain a better understanding of the complex physical mechanisms that take place during the solidification process. This in turn will help the food engineers to improve the processing, storage, quality as well as the shelf life of the products. A complete literature review of the detailed numerical approach is presented by Delgado and Sun [3].

While a comprehensive numerical model can capture the crystallization physics more accurately than a simple algebraic model, it is also computationally expensive. In fact, the application of such a model to several thousands of droplets in a spray system is not computationally feasible. On the other hand, the simple algebraic formulation of Plank is fast, but of little use in understanding the crystallization characteristics in detail. In an attempt to meet the dual objective of low computational stress as well as a reasonably elaborate representation of the solidification process, an intermediate numerical model is presented in this study. The model is primarily applicable for particle type materials that are subject to spray crystallization.

Spray crystallization is a process in which liquid droplets are sprayed into a cold ambient environment to crystallize into a powder [4]. It has a direct application in industries such as food products [5,6], ceramics [7], metallurgy [8], biology [9], pharmaceuticals [10,11,12,13,14] and artificial snow production [15] where spray crystallization has been successfully employed. This approach of powder production has the following advantages: 1. End products are stable as well as thermally reliable. 2. Due to the high surface to volume ratio, fast cooling rates via rapid convective heat transfer can be achieved. Additionally, in case of volatile solutions, the cooling rate is further enhanced due to the mass transfer in which some water content is removed via evaporation. 3. With small droplet sizes a homogeneous temperature field for solidification can be provided which will yield a powder with a uniform micro-structure. Thus, with spray crystallization, we can get final powder products with a uniform size distribution. 4. Finally, this approach does not require the need to separate the solids from the liquid phase of the solution before the mixture is subjected to crystallization.

Meryman [16] introduced this technique to the food industry. Subsequently, it has been studied by several researchers to obtain powder forms of food products. For instance, Hindmarsh et al. [17] conducted experimental investigations and performed numerical simulations to understand the freezing of sucrose solution droplets. The authors presented the freezing of a sucrose droplet as a five-stage process: Stage one is comprised of the *liquid cooling and supercooling*, in which the droplet cools from its initial state to a temperature below the equilibrium freezing point. At this point, the second stage, i.e., *nucleation*, is initiated. This instantaneous phenomena causes the initiation of the crystal nucleus formation. This results in the third stage of *recalescence* where there is a sudden rise in the temperature of the droplet to the equilibrium freezing temperature, $T_{f}$. This increase is due to the release in the latent heat during the crystallization process. It is followed by the fourth stage of *freezing* during which the droplet undergoes a gradual phase change until it is completely frozen. Finally, in the fifth stage there is a cooling of the solid droplet, where its temperature reduces to that of the ambient air. The numerical results based on this model agreed well with their experimental freezing profiles.

In another study, Hindmarsh et al. [18] applied the numerical model to the experimental investigation of the freezing of water and sucrose solution droplets. The authors used Nuclear Magnetic Resonance (NMR) spectrometry to obtain the experimental values. They found that the agreement between the numerical calculations and the experimental results were good for water. However, for the sucrose solution droplets, the agreement was only good when the ambient air temperature was relatively high. They attributed the discrepancies at the lower temperatures to variations in the actual crystallization temperatures.

Macleod et al. [19] investigated the freezing of instant coffee droplets using NMR techniques. They compared these results with the ones obtained via numerical modeling. From their heat transfer measurements and computational results, they concluded that the internal temperature of the droplets were almost uniform. This was also in agreement with their observation on the formation of the micro-structures.

It should be noted that a five-stage freezing behavior is not seen in all substances. For instance, the presence of undercooling is largely dictated by the number of nucleating agents. Specifically, if there is a high degree of impurity in the solution, i.e., many nucleating agents, then crystallization is initiated as soon as the phase change temperature is reached, e.g., cocoa butter. Thus, such substances do not experience undercooling. Likewise, phase change can occur at a constant temperature or over a range of temperatures, and is dictated by the amount of impurities in the solution. Thus, pure water freezes at a constant temperature [20]. On the other hand, one can see a dip in the freezing temperature, known as the freezing point depression, due to the presence of impurities, e.g., sucrose solution [18] or coffee solution [19]. In fact, in substances like cocoa butter, the phase change takes place over a range of temperatures without any undercooling. Thus, from a temperature profile of such substances one can subdivide the entire solidification process into just three stages, viz., pre-cooling, solidification and tempering.

Our interest is in understanding the solidification associated with spray crystallization employed in the food sector, confectionery industry in particular. In this industry, there is a significant interest in understanding the crystallization of cocoa butter (CB) in the chocolate manufacturing process [21,22,23,24,25]. This is because CB fat powders are used as an ingredient in chocolate or are used as a seed powder for crystallization while processing chocolate masses. For such purposes, with a clearly defined crystalline structure of CB powder, better products with improved qualities such as storage stability, melting behavior, consistency, etc., can be obtained [5]. Therefore, as a preliminary step towards understanding the crystallization process of cocoa butter in greater detail, a numerical model that is capable of emulating the solidification process of particles is presented in this study.

The model consists of the following stages: The first stage is the pre-cooling stage where the droplet temperature decreases as per a simple convective heat transfer model. Once the droplet reaches the phase change temperature, $T_{f}$, the second stage (solidification), where the droplet begins to solidify, is initiated. When the phase change period is completed, the cooling of the solidified droplet (stage three) starts. Here, the temperature of the droplet decreases gradually to a steady state value near that of the ambient air.

The model has been applied to simulate the solidification of a pure CB droplet without any water content and is validated using the experimental data of Gwie et al. [26]. For this, the model was implemented into the three-dimensional Fortran-based computational fluid dynamics (CFD) code, *KIVA-3*. As part of model validations, CFD simulations were made in which a single 2 mm CB droplet was suspended in a cylindrical chamber with inflow–outflow boundary conditions. More precisely, there was an inflow of cold air from a narrow hole at the center of the bottom of the cylinder. The top was configured as an open boundary. The droplet, suspended very close to the inlet hole, experiences the cold air and solidifies over time. This is similar to the experimental set up of Gwie et al. [26]. The implementation and validation of the solidification model are discussed in the ensuing sections.

A key highlight of this manuscript is the utilization of the model to study various process conditions. Specifically, the model has been applied to understand the crystallization time of a single CB droplet for various process conditions. These simulations were made as a prelude to a more thorough optimization study. In these, the impact of ambient air temperature and the relative drop-gas velocity on the solidification process of droplets of different sizes were investigated. In the CFD studies, single droplet solidification simulations were undertaken in which the droplet was subject to various combinations of two different ambient temperature and relative drop-gas velocity conditions. Further details are presented in the section titled *Parametric Study of the Crystallization Process*.

## 2. The Three-Stage Solidification Model

The temperature change in a material can be represented as being governed by the internal heat conduction as well as a forcing term that accounts for the internal heat generation and boundary conditions. Thus, the temperature distribution inside the material at any instant can be obtained as the solution to the following equation:

(1) $$\rho C_{p}\frac{\partial T}{\partial t}=\nabla \cdot \left(k\nabla T\right)+S.$$

In the above equation ρ, $C_{p}$, *T*, *k* and *S* are the density, the specific heat capacity, temperature, the thermal conductivity of the material and the forcing term, respectively.

In the absence of any internal heat generation and assuming a convective heat flux through the surface of the material, the forcing term (*S*) can be modeled via the following equation:

(2) $$S=Ah(T_{a}-T_{d})/V,$$

where *A* and *V* are the surface area and volume of the material, respectively. For a spherical droplet of diameter *d*, considered in this study, these would be $A=\pi d^{2}$ and $V=\pi d^{3}/6$. Additionally, $T_{a}-T_{d}$ represents the difference between the ambient air temperature ($T_{a}$) and the droplet surface temperature ($T_{d}$). Thus, Equation (1) can be written as

(3) $$\rho C_{p}\frac{\partial T}{\partial t}=\nabla \cdot \left(k\nabla T\right)+\pi d^{2}h\left(T_{a}-T_{d}\right)/\left(\pi d^{3}/6\right).$$

For the heat flux through the surface, the heat transfer coefficient, *h*, is evaluated using the Ranz–Marshall correlation as [27]

(4) $$Nu=\frac{hd}{k_{a}}=2+0.6Pr^{1/3}Re^{1/2},$$

where Nu is the Nusselt number and $k_{a}$ is the thermal conductivity of air. Pr and Re are the Prandtl number and Reynolds number, respectively, that are given by the following relations:

(5) $$\begin{array}{ccc} Re & = & \frac{d\rho_{a}V_{dg}}{\mu_{a}},\\ Pr & = & \frac{C_{p}\mu_{a}}{k_{a}}. \end{array}$$

In the above equation, $\rho_{a}$ is the ambient air density, $V_{dg}$ is the relative droplet-gas velocity and $\mu_{a}$ is the viscosity of the surrounding air. From Equations (4) and (5), it is clear that the heat transfer coefficient, *h*, is not a constant since it is coupled to the ambient gas properties ($k_{a}$ and $\mu_{a}$) that change with time in the immediate neighborhood of the droplet.

Now, for small droplets with low Biot numbers (typically less than 0.1) the internal heat conduction is very fast compared to the convective heat transfer from their surface. In other words, the temperature gradients (∇T) inside the droplet are negligible, i.e., at any given time a uniform droplet temperature, equal to the droplet’s surface temperature ($T_{d}$), can be assumed. Thus, Equation (3) can be simplified further as

(6) $$\pi \frac{d^{3}}{6}\rho C_{p}\frac{\partial T_{d}}{\partial t}=\pi d^{2}h\left(T_{a}-T_{d}\right).$$

The Biot number (Bi), that is used as a measure for this approximation, is defined as the ratio of the measure of convection to conduction and is evaluated as

(7) $$Bi=\frac{hl}{k}.$$

In this equation, *h* is the heat transfer coefficient, *l* is the characteristic length and *k* is the thermal conductivity. Further, the characteristic length, *l*, is the ratio of the volume of the body to the body’s area that is perpendicular to the direction of heat flow. In order to justify the uniform droplet temperature assumption an upper limit of 0.1 on the Biot number is usually used as a criterion [28]. For micron sized CB droplets this criterion is satisfied with $Bi\approx O(10^{-2})$ and hence the uniform droplet temperature assumption.

Equation (6) forms the basis for the solidification model presented in this study. In presenting this model, in addition to the above simplifications, two assumptions have been made: (1) The thermophysical properties of the material are constant in the solid and the liquid states. However, they might be different in the two states. (2) Additionally, it is assumed that there is no mass exchange between the droplet and the ambient air. In view of the fact that the target droplet size in the spray system are in the micron range where the mass transfer rates are much smaller than the convective heat transfer, this assumption is reasonably accurate. Thus, in our simulations, the diameter of the droplets are constant. This assumption has also been used by other researchers in studying the solidification of droplets [19,26]. The three-stage model incorporating these assumptions is then described as follows:

**Stage 1:** This is the pre-cooling stage where the liquid droplet starts to cool from its initial temperature ($T_{ini}$) to the phase change temperature ($T_{f}$). Accordingly, the heat capacity of the liquid material ($C_{p_{d}}$) is used in Equation (6). The rate of change of the droplet temperature in this stage is modeled as:

(8) $$\frac{\pi d^{3}}{6}\rho C_{p_{d}}\frac{dT_{d}}{dt}=\pi d^{2}h\left(T_{a}-T_{d}\right).$$

It should be noted that the droplet size is assumed to be constant in all the three stages, i.e., there is no change in the mass or the volume of the droplet. Hence, the density, ρ, is the same in all three stages. Since the droplets are very small, using a constant density is justified.

**Stage 2:** The solidification stage is initiated once the droplet reaches the phase change temperature, $T_{f}$. For CB this temperature has been chosen according to Loisel et al. [24] as $T_{f}=291$ K. In our model, we assume that the droplet starts solidifying from the outer surface towards the center. Further, since the heat capacity of a liquid and solid CB are different, it is evident that the semisolid droplet cannot have a constant heat capacity. In fact, this value is likely to change based on the solidified volume fraction of the droplet. In order to take this into account, the heat capacity of the semisolid is linearly varied from $C_{p_{d}}$ (specific heat capacity of liquid CB) to $C_{p_{s}}$ (specific heat capacity of the solid CB) during this stage. More precisely, the heat capacity of this phase, $C_{p_{sd}}$, is based on the solidified depth of the droplet and is given by the following relations:

(9) $$\begin{array}{ccc} a & = & \frac{r_{l}}{r}, \end{array}$$

(10) $$\begin{array}{ccc} C_{p_{sd}} & = & \left(1-a\right)C_{p_{s}}+aC_{p_{d}}, \end{array}$$

where *a* is a progress variable that evolves from 1 to 0 as the droplet begins to solidify, *r* is the radius of the droplet and $r_{l}$ is the radius of the liquid portion of the droplet. At the start of solidification when $T_{d}=291$ K the entire droplet is in the liquid state, i.e., $r_{l}=r$, the radius of the droplet. On the other hand, when the droplet is completely solid $r_{l}=0$ and a=0. Thus, the completely solid droplet has a heat capacity of 1.25 kJ/kgK.

On a graph of the temperature profile, the beginning of stage two is identified by a sharp decrease in the rate of change of temperature of the droplet. This is because during the crystallization process there is a release of the latent heat while the atoms and molecules are rearranging themselves to form a crystalline structure. This will contribute to the heating of the droplet. This source term is incorporated into Equation (8) and the solidification stage is given as

(11) $$\frac{\pi d^{3}}{6}\rho C_{p_{sd}}\frac{dT_{d}}{dt}=\pi d^{2}h\left(T_{a}-T_{d}\right)+\rho {\dot{V}}_{f}L.$$

In the above equation $C_{p_{sd}}$ is the heat capacity of a semisolid droplet that is given by Equation (9), ${\dot{V}}_{f}$ is the volumetric solidification rate and *L* is the latent heat of crystallization that has an approximate value of 157 kJ/kg for CB [29].

**Stage 3:** Once the droplet is completely solid, it continues cooling as per the heat transfer model described in Equation (8). However, this time $C_{p_{s}}$, i.e., heat capacity of the solid CB, is used instead of $C_{p_{d}}$. In the temperature profile of the droplet, the initiation of this stage can be observed as a distinct increase in the rate of decrease of the temperature. The cooling process continues until the droplet reaches the ambient temperature.

An important feature of the solidification model just described is that the latent heat capacity and the specific heat capacity have been decoupled in the solidification stage and appear separately in Equation (11). An alternative approach is to use the *apparent specific heat* ($C_{p_{app}}$) in a single equation (Equation (8)) for all three stages. For this, using the experimental data on the heat transfer from the material to the surrounding, the apparent specific heat over a range of temperatures is specified. Thus, $C_{app}$ has the latent heat merged with the specific heat to produce a large peak in the range of temperatures over which solidification occurs. More precisely, a typical graph of $C_{p_{app}}$ versus temperature will have the following characteristics: At temperatures above the solidification point, $C_{p_{app}}$ is equal to the specific heat capacity of the liquid form of the material ($C_{p_{d}}$). Likewise, in the tempering stage $C_{p_{app}}$ is equal to $C_{p_{s}}$, the heat capacity of the solid material. In the range of temperatures over which solidification occurs there will be a sharp increase in $C_{p_{app}}$ to account for the latent heat that is released during the phase change period. Thus, depending upon the material, this part of the graph is a spike or a smooth bell-like shape. The need of experimental data to formulate the model prevents the easy application of the model.

As an alternative to a database of $C_{p_{app}}$ at various temperatures for a particular material, researchers also use analytical expressions that are derived from the analysis of the database to estimate $C_{p_{app}}$ at a particular temperature. Tavman et al. [30] have summarized such equations that approximate $C_{p_{app}}$ as a function of temperature for meat and shrimp products, as presented by several researchers. Nevertheless, these formulations still rely on experimental data for obtaining the constants in the equations. On the other hand, in the present approach just the knowledge of the specific heat capacity at the solid and liquid state, and the latent heat capacity of the material is sufficient to apply the model. Further, it must also be mentioned that the decoupling technique used here is more reliable than using apparent specific heat approach as is discussed next.

By using $C_{p_{app}}$, there is a high probability of underestimating the latent heat if the temperature at a computational node over steps to miss the peak of the $C_{p_{app}}$ versus temperature curve. Specifically, in materials where the latent heat is released over a very short temperature range it is easy to miss the apparent specific heat capacity spike and the consequent underestimates can lead to large errors. This has already been pointed out by Pham [31] as well as Cleland and Warle [32]. This is not the case in the present approach in which the solidified fraction and the amount of latent heat released are closely coupled. More precisely, a small increment in the solidified volume fraction ($\Delta V_{f}$) is accompanied by the release of an equivalent amount of latent heat (ΔL), which helps avoid these errors. Thus, a complete solidification is accompanied by the release of all the latent heat.

Finally, a significant advantage of the model is its applicability to a system with several thousands of droplets. This is mainly attributed to the uniform droplet temperature assumption which simplifies the model greatly. This assumption results in a reasonably small computational time for a single droplet. Thus, in a spray system with thousands of droplets, it is computationally feasible to apply this model to every droplet in the system. As mentioned earlier, since the droplets of interest are micron-sized, the Biot numbers are small enough to assume a uniform droplet temperature. Put differently, the accuracy of the model is not compromised by the simplified model.

## 3. Computational Aspects and the Numerical Scheme

Modeling the transient three-dimensional dynamics of the sprays and their interaction with the gas phase requires a complex code. *KIVA-3* [33], a Fortran-based CFD code used in this study, is equipped with advanced models that are capable of simulating these multi-phase flows and the interaction between them [34,35,36,37,38,39,40,41]. For the gas phase, *KIVA-3* includes a complete set of transport equations, including the mass, momentum, energy and the species equations [42,43]. Additionally, there are two transport equations for the turbulent kinetic energy and its dissipation rate, to model the turbulence in the computational domain. For the dispersed liquid phase, *KIVA-3* is equipped with a spray evolution equation. This is a transport equation that is solved to obtain an accurate distribution of the droplets’ sizes, velocities and temperatures.

When liquid drops are injected into the system, there is a continuous interaction between the liquid and the gas phase via a constant exchange of momentum and energy between them. This coupling between the two phases is accomplished via the source terms in the governing equations of the gas phase. *KIVA-3* has models to compute these source terms. Additionally, there are models to account for the interaction within the liquid phase, such as collisions, coalescences and break up. Further details can be found in the Ph.D. Thesis of Srinivasan [44].

The three-stage solidification model, described in the previous section, has been implemented in *KIVA-3*. The coupling between this model, that essentially works on the droplets, and the ambient gas has been accomplished as follows: At each time instant when the droplet rejects heat to the cold ambient air, the domain’s internal energy is updated (proportionally increased) at the location of the droplet. It should be noted that this change in the internal energy effects the air properties (viscosity and thermal conductivity) in the immediate neighborhood of the droplet. As a consequence there is a marginal change in the heat transfer coefficient (cf. Equations (4) and (5)) that in turn impacts the rate of change of the droplet temperature in each stage.

In the numerical scheme, a finite volume approximation of the complete set of governing equations has been solved. For this, the computational region is subdivided into hexahedral cells, which form the computational mesh. The integral form of the transport equations are then discretized in both time and space. The temporal differencing is performed with respect to a sequence of discrete times, $t^{n}$. The time intervals, $\Delta t^{n}=t^{n+1}-t^{n}$, is the time step size in the *n*th cycle. Thus, a time marching scheme is used in which the dependent variables in each time step is determined from the values in the previous time step. The spatial differencing is obtained by writing the difference equation with respect to vertices of the cells of the computational mesh. A central differencing scheme is used for the diffusion terms and a forward difference scheme is used for the convection terms. Discretization of the liquid phase is done using a stochastic particle technique in which the droplet distribution, *f*, is discretized by means of particles or parcels [33].

The solution of the discretized equations is based on the the Arbitrary Lagrangian Eulerian (ALE) method. A cycle in *KIVA-3* is performed in three phases. In phase A, the spray droplet collision and oscillation/breakup terms are computed. Additionally, the solidification model is applied to each droplet in the system. In this, for every droplet in the system the appropriate equation of the three-stage solidification model is implicitly solved to obtain the new droplet temperature. Following this, the internal energy of the cell in which the particle is located is immediately updated based on the amount of heat rejected by the droplet.

In phase B, the pressure gradient in the momentum equation, the velocity dilation terms in the mass and energy equations, the spray momentum source term and the terms due to the diffusion of the mass, momentum and energy are calculated. Phase B also calculates the remaining source terms in the turbulence equations. These flow field variables are computed by solving the implicit equations of phase A. The solution procedure is based on a SIMPLE (Semi Implicit Method for Pressure Linked Equations) type algorithm. It should be noted that in this study, there is no turbulent motion inside the chamber and so the turbulence model has been deactivated in the simulations.

Finally, in phase C, the convection terms are calculated. This is done in a sub-cycled, explicit calculation using a time step, $\Delta t_{c}$, that is a fraction of the original time step Δt. A quasi-second order upwind (QSOU) differencing is used for solving the convective terms. Further details of the numerical procedure can be found in [33].

## 4. Model Validation

In this section, the validation of the solidification model for a single CB droplet is presented. The validation has been done using the experimental data of Gwie et al. [26]. The thermophysical properties of CB that have been used for this are summarized in Table 1. For the model validation a fixed cylindrical domain with a diameter of 2 cm and a height of 1 cm has been used. There is an inflow of ambient air through a hole of diameter 0.32 cm at the center of the bottom face of the cylinder. The top face of the cylinder is configured as an open outflow boundary. For the computational domain, a structured, hexahedral, polar mesh with 12×20×10 cells, uniformly distributed in the radial, azimuthal and axial directions, respectively, has been used. This translates to a constant resolution of 0.83 mm and 1 mm along the radial and axial directions, respectively.

The baseline case that has been simulated involves a single 2 mm CB droplet that has been suspended along the axis at a distance of 0.2 cm from the bottom of the cylindrical domain. Further, this initial position of the droplet is held constant during the entire simulation. The initial temperature and pressure of the domain are set at 277 K and 1 bar, respectively. Air at 277 K and with a velocity of 83 cm/s is continuously injected through the inflow boundary. Thus, as in the experimental study, the droplet experiences a relative (drop-gas) velocity due to the air flow from the hole at the bottom of the cylinder. The details of the experimental conditions of Gwie et al. [26] are summarized in Table 2.

It must be stated that the choice of a 2 mm droplet is purely because of the fact that the available experimental data is for a droplet of this size. The Biot number of a 2 mm CB droplet is approximately 0.18. This is clearly higher than the generally prescribed upper limit of 0.1. However, other researchers have used a similar numerical model with even higher Biot numbers and have reported good agreement with the experimental data [19,26].

**Figure 1:**
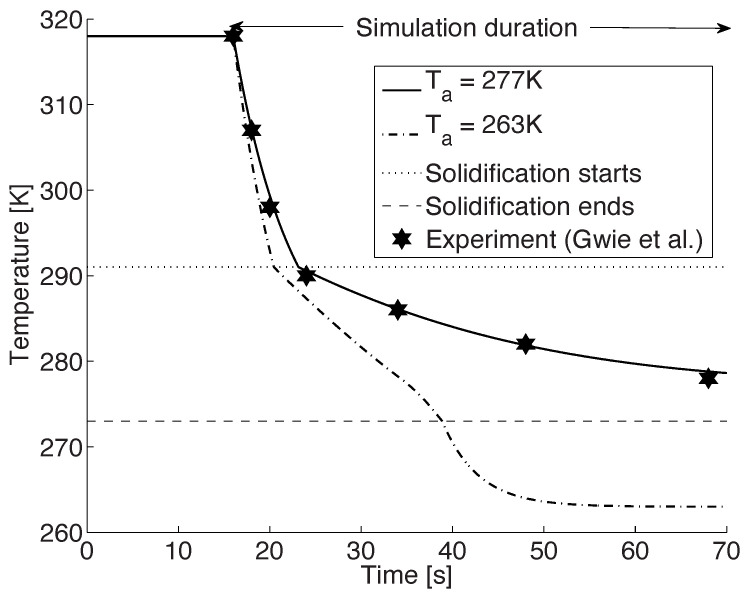
Temperature profile of a 2 mm droplet, initially at 318 K, suspended in a chamber that is at 277 K and 1 bar, along with the experimental data of Gwie et al. [26] (asterisk symbol, copyright permission has been obtained). An additional simulation is performed at $T_{a}=263$ K. The start of solidification is at 291 K and the solidification ends at 273 K. (Figure reproduced from [42,43] by resimulating the model).

Figure 1 shows the computed cooling profile of the CB droplet in the baseline case ($T_{a}=277$ K) along with the experimental values of Gwie et al. [26]. As is evident, there is an excellent agreement between the two. In the first stage, the droplet undergoes a rapid initial cooling due to a convective heat transfer. Starting from an initial temperature of 318 K the droplet cools at a rate of approximately 6.3 K/s in this pre-cooling stage and in about 7 s it reaches the phase change temperature of 291 K. This cooling rate is in close agreement with the experimental values which lie in the range 7–9 K/s.

Upon reaching this phase change temperature of 291 K, the solidification of the CB droplet is initiated. This is observed as an abrupt change in the rate of decrease of temperature (cf. Figure 1). During this stage, the droplet cools with an initial cooling rate of about 0.5 K/s. Thus, after about 48 s of start of solidification, the temperature of the droplet is 277 K. The average values of Nusselt number and the heat transfer coefficient for this process are approximately equal to 7.85 and 96.86 W/m^2^K, respectively. Again, these are in good agreement to the estimates of Gwie et al. [26]. It must be noted that in our model, the Nusselt number as well as the heat transfer coefficients are closely coupled to the ambient air properties, viz., $\rho_{a}$, $\mu_{a}$ and $k_{a}$ (cf. Equations (4) and (5)). On the other hand, Gwie and co-workers have used the following simple correlations to estimate the heat transfer coefficient and the Nusselt number from their experimental measurements of $T_{d}$

(12) $$\begin{array}{ccc} h & = & \frac{C_{p}\rho d\ln\left(T_{d}-T_{a}\right)}{6t}, \end{array}$$

(13) $$\begin{array}{ccc} Nu & = & hd/k_{a}. \end{array}$$

In the above equation, *t* is the solidification time of the droplet. The differences in the estimates of Nu and *h* between our work and their results are a direct consequence of this. The simulation results and their comparison with the estimates from the experimental data are summarized in Table 3.

The change in the rate of decrease of temperature at the solidification temperature of 291 K is due to two competing phenomena. In the pre-cooling stage, there is a continuous transfer of heat from the warm droplet to the environment. However, as crystallization begins at 291 K, there is a release of latent heat that will contribute towards the increase of the droplet temperature. Nevertheless, since the rate of decrease of temperature is greater than the contribution due to latent heat, there is a net decrease in the temperature of the droplet, but at a subdued rate.

In the experimental investigations by Gwie et al. [26], the authors found that even after 48 s of entering the second stage, the CB droplet, which was now at 277 K, had not completely solidified. In general, when solidification is complete there is no more latent heat released to counter the cooling process. As a result, the cooling should proceed without any hindrance and would be indicated by a sudden increase in the rate of fall of temperature. This expected change, however, was neither observed in the experiment, nor in the simulation.

In order to observe this transition and to ensure that all the stages of the solidification process were initiated appropriately, the simulation was repeated with the ambient temperature reduced to $T_{a}=263$ K. More precisely, a freely suspended 2 mm droplet at an initial temperature and pressure of 318 K and 1 bar, respectively, was subjected to an ambient temperature of 263 K. At the inlet boundary, air at a temperature of 263 K entered the system with a velocity of 83 cm/s. Figure 1 shows the temperature profile of the droplet from this simulation ($T_{a}=263$ K). After the pre-cooling and solidification stage when the droplet reaches a temperature of 273 K, it is completely solid. Beyond this point the tempering stage of the model starts in which the solid droplet cools to the ambient temperature. The initiation of this stage is observed as a higher rate of decrease of the temperature at $T_{d}=273$ K at about t=38 s (cf. Figure 1).

It must be mentioned that the choice of 273 K as a temperature at which CB is assumed to be in a solid phase is based on the following arguments: Researchers have observed that crystallization of CB can occur over a wide range of temperatures, typically from 263 K to about 298 K [24,45]. Additionally, various forms of crystalline CB can be realized based on the process conditions. Further, depending upon the size of the sample being considered, some of these crystalline forms can be obtained from the initial liquid state in as early as a few seconds while others could take as long as a few weeks [45]. Since the regime of interest in our future spray investigations will be in the micron range, the assumption that the CB droplets would be in a solid state at 273 K is reasonable.

On comparing the temperature profiles of the two simulations we can see that there is a large difference in the cooling rates in the first two stages (cf. Figure 1). More precisely, the cooling rates in the lower ambient temperature case are larger. Additionally, in this simulation, once the droplet is completely solidified at about 38 s, we observe an increase in the rate of fall of the droplet temperature and the solidified droplet quickly reaches the ambient temperature.

It should be noted that these differences in the cooling pattern are due to the fact that the rate of decrease of the temperature in any stage is directly proportional to the temperature difference between the droplet and the ambient air. In other words, these variations can be attributed to the term ($T_{a}-T_{d}$) in Equations (8) and (11). With an increase in this term, the cooling rates are expected to accelerate, i.e., the colder the domain is, the faster the droplet cools and solidifies.

## 5. Mesh Resolution Analysis

In order to ensure that the above results are independent of the mesh size, a mesh resolution analysis was performed. For this, starting from the standard mesh of the baseline case, a relatively coarser mesh was obtained by reducing the number of cells in all three direction by a factor of 2. Thus, the coarse mesh had 6×10×5 cells uniformly distributed in the three respective directions. For a cylindrical domain of height 1 cm and diameter 2 cm, this corresponds to a constant resolution of 1.7 mm and 2 mm along the radial and axial directions, respectively. The fine mesh for this domain had twice the number of cells in the standard mesh, i.e., 24×40×20 cells in the three directions. Again, the cells were uniformly distributed in the domain. In other words, along the radial and axial directions, this mesh had a fixed resolution of 0.4 mm and 0.5 mm, respectively.

**Figure 2:**
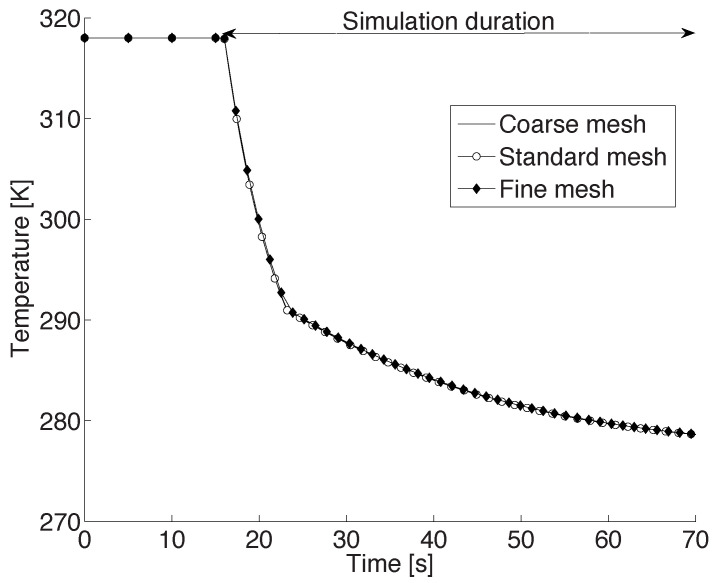
Temperature profile of a 2 mm droplet, initially at 318 K, suspended in a chamber that is at 277 K and 1 bar, using three different mesh resolutions.

For the mesh resolution analysis, the baseline case was repeated, i.e., a 2 mm CB droplet was suspended along the axis of the cylinder at a height of 0.2 cm from the bottom of the cylinder. Further, air at 277 K with a velocity of 83 cm/s was blown from the narrow inlet hole at the center of the bottom of the domain. The computations were made until the droplet temperature reached the ambient air temperature. The transient temperature profile of the droplet as computed using the three meshes are presented in Figure 2. As seen in this figure, the temperature profiles are almost indistinguishable, indicating an excellent mesh independence. In other words, a standard mesh gives adequately accurate results. In the ensuing section, this mesh is therefore used to investigate the impact of the process conditions on the solidification behavior of a CB droplet.

## 6. Parametric Study of the Crystallization Process

The crystallization behavior of the spray droplets is expected to depend on the processing conditions such as the the spray parameters and the chamber properties. For instance, by adjusting the spray characteristics one can influence the droplet sizes and the droplet velocities, i.e., the relative drop-gas velocity ($V_{dg}$). These changes will reflect on the solidification behavior via the heat transfer coefficient. Likewise, changes in the chamber properties such as the ambient temperature can impact the rate of change of the droplet temperature and thereby the time scales of the powder production process.

In general, it is desirable to have a very rapid crystallization, i.e., a short crystallization time. However, this means higher energy requirements, which is not a favorable option. Hence, to address this trade-off it is important to understand the crystallization behavior in relation to the process parameters. A subsequent optimization would then help us determine a suitable operating condition to minimize the energy requirements while maintaining a reasonably small crystallization time. As a prelude to a detailed optimization study, the effect of the ambient temperature ($T_{a}$) and the relative drop-gas velocity ($V_{dg}$) on the crystallization time of droplets different sizes have been investigated. More precisely, simulations pertaining to four different drop sizes are presented, viz., 200, 300, 400 and 500 microns. Further, for each size, two different ambient temperatures and relative drop-gas velocities were considered. Specifically, $T_{a}$ of 220 K (low $T_{a}$) and 270 K (high $T_{a}$), and $V_{dg}$ of 20 cm/s (low $V_{dg}$) and 100 cm/s (high $V_{dg}$) were used. In all the simulations, the ambient air was at a pressure of 1 bar. The computational set up in each simulation, including the boundary conditions, was as in the baseline case presented in the Section 4.

**Figure 3:**
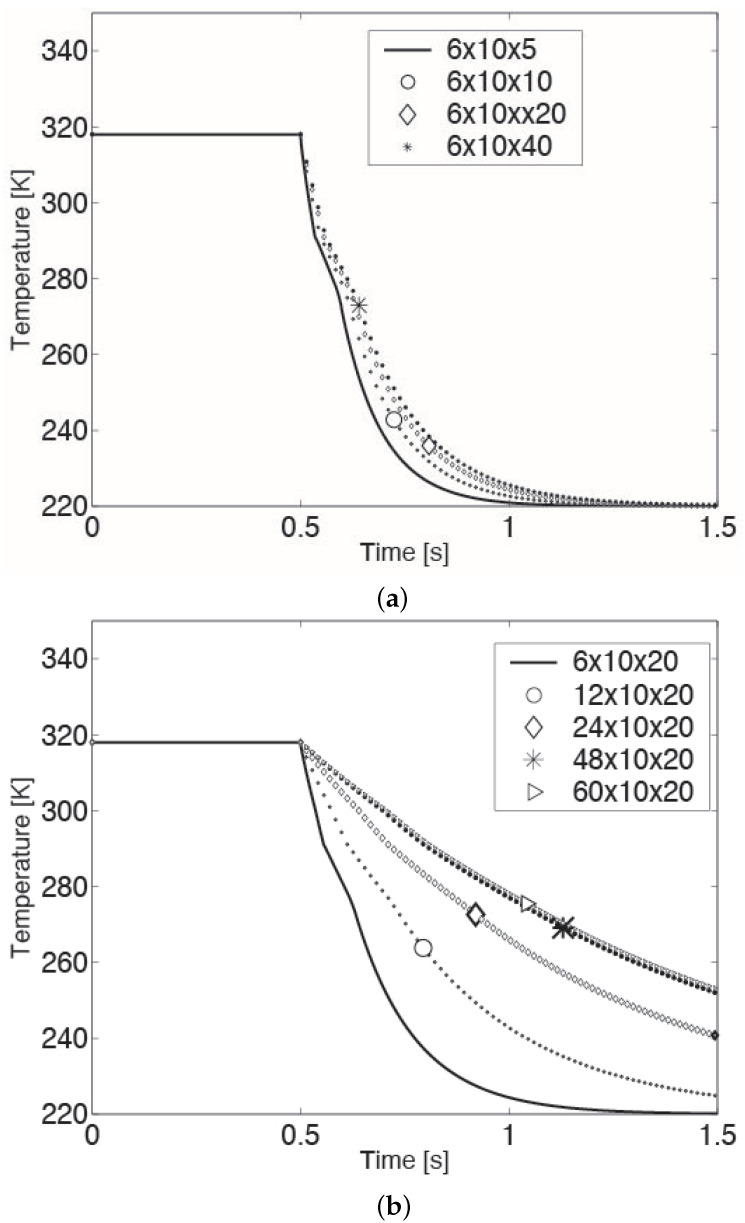
Mesh refinement along the (**a**) axial and (**b**) radian directions for a 100 micron droplet experiencing an ambient temperature and relative drop-gas velocity of 220 K and 20 cm/s, respectively.

It must be noted that unlike the model validation case, the droplet size in this parametric study is an order of magnitude smaller. Hence, before conducting the above simulations, to ensure mesh independence, a mesh refinement was done for a 100 micron CB droplet experiencing an ambient temperature of 220 K and a relative drop-gas velocity of 20 cm/s. Specifically, starting from a grid with 6×10×5 cells uniformly distributed in the radial, azimuthal and axial directions, respectively, refinement was done in each direction until a reasonable mesh independence was obtained. More precisely, the mesh was first refined in the axial direction, keeping the number of cells in the radial and azimuthal directions fixed (cf. Figure 3a). This yielded a mesh independence in the axial direction with a grid of 6×10×20 cells in the radial, azimuthal and axial directions, respectively. In Figure 3a, the relative error between the 6×10×20 mesh and 6×10×40 mesh is less than 2%. Next, this procedure was applied along the radial direction, retaining the refinement (20 cells) in the axial direction (cf. Figure 3b). The end result was a mesh with 48×10×20 cells in the radial, azimuthal and axial directions, respectively, with grid independence in the radial as well as axial directions. The relative error between the 48×10×20 mesh and 60×10×20 mesh was less than 1%. Finally, a refinement in the azimuthal direction was pursued, keeping the number of cells in the radial and axial directions at 48 and 20 cells, respectively. It was found that the temperature profile of the droplet was almost impervious to changes (increments) in the number of cells in this direction. Hence, a mesh with 48×10×20 cells in the three respective directions was used in the parametric study.

**Figure 4:**
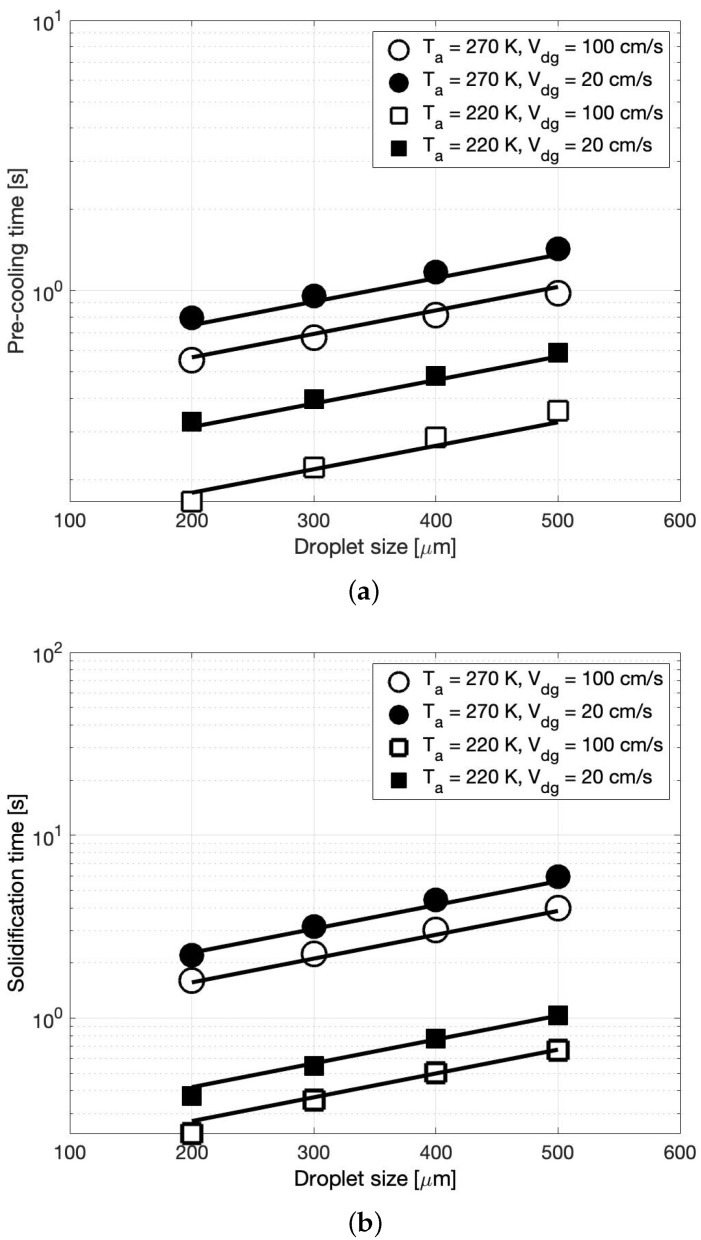
CFD simulation estimates of (**a**) the pre-cooling time and (**b**) the solidification time, as a function of the droplet size for several combinations of ambient temperature and relative drop-gas velocity (symbols). Trendlines (solid line) in (**a**) is as per Equation (14) and in (**b**) is as per Equation (15).

Figure 4a,b summarize the pre-cooling and solidification time, respectively, as a function of the droplet size for all four combinations of ambient temperature and relative drop-gas velocity. In these figures, the circle and square symbols represent the ambient temperatures of 270 K and 220 K, respectively. The open and closed symbols represent a relative drop-gas velocity of 100 cm/s and 20 cm/s, respectively. As seen in Figure 4a, for each drop size, as the ambient temperature decreases, the droplet cools to the solidification temperature of 291 K faster. This behavior is true at both values of $V_{dg}$. An analogous observation can be made for the solidification time (cf. Figure 4b), i.e., as the ambient temperature increases, the time needed by the droplets to solidify increases. This behavior is explained by the fact that with an increase in the relative temperatures between the droplet and the ambient air, the droplet experiences a rapid decline in its temperature (cf. Equations (8) and (11)). In other words, the droplet will cool faster. Thus, it is desirable to have a low ambient temperature to obtain short crystallization times.

With respect to the relative drop-gas velocity, it is seen that at a given ambient temperature, an increase in $V_{dg}$ decreases the time needed for the droplet in each stage. Conversely, for a short crystallization time it is desirable to have a high relative drop-gas velocity. This is because a larger $V_{dg}$ means higher Reynolds number and thereby a larger heat transfer coefficient (cf. Equations (4) and (5)) that would result in faster cooling. Finally, a comparison of the effects of $T_{a}$ and $V_{dg}$ indicates that the precooling as well as the solidification time are more sensitive to the changes in $T_{a}$ than to the changes in $V_{dg}$. In other words, $T_{a}$ has a dominant impact on the crystallization time. This is evident in these figures from the larger variations in the pre-cooling as well as solidification time for changes in $T_{a}$ than for changes in $V_{dg}$. This is expected since the rate of change of the droplet temperature is proportional to $\left(T_{a}-T_{d}\right)$ and is only impacted by the square root of $V_{dg}$ (cf. Equations (4) and (5)).

In general, from Figure 4a,b we can see that the time in each stage increases exponentially with the droplet size. In fact, following a least squares analysis of the CFD simulation points in these figures, it was found that the pre-cooling ($t_{\mathrm{pre}-\mathrm{cooling}}$) and solidification time ($t_{\mathrm{solidification}}$) in the four $T_{a}-V_{dg}$ combinations were related to the droplet size as

(14) $$t_{\mathrm{pre}-\mathrm{cooling}}=A\exp\left(0.002d\right),$$

(15) $$t_{\mathrm{solidification}}=B\exp\left(0.003d\right).$$

In the above equations, *d* is the droplet size in microns, and *A* and *B* are constants that are summarized in Table 4. It must be noted that the larger exponent in expression for $t_{\mathrm{solidification}}$ reflects the fact that during solidification process the latent heat that is released tries to increase the droplet temperature, thereby retarding the rate of decrease of the temperature, i.e., increasing the time in stage two. Additionally, it is interesting to observe in Table 4 that at a high ambient temperature (270 K), *B* is slightly larger than 2A, whereas at a low ambient temperature *B* ≈ *A*. These trends in Equations (14) and (15) are represented in Figure 4a,b, respectively, using solid lines. As can be seen from these figures, the correlations are in good agreement with the data from the CFD simulations.

## 7. Conclusions

A major utility of our investigation is in determining the appropriate environment conditions in a production facility to achieve the desired end product with minimum operational costs that are incurred in maintaining a certain ambient temperature, ambient air velocity, etc. Several CFD simulations, employing an experimentally validated multi-stage solidification model, were made to understand the influence of ambient temperature and the relative drop-gas velocity on the crystallization behavior of micron-sized cocoa butter droplets. It was found that in general, for any combination of $T_{a}$ and $V_{dg}$, the pre-cooling as well as the solidification times increased exponentially with the drop size. This increase is because a larger droplet means more heat has to be removed before it completely solidifies. Further, an increase in $T_{a}$ results in an increase in the crystallization time. This is attributed to a small drop-gas temperature difference, which will reduce the cooling rate. Finally, higher $V_{dg}$ means larger Reynolds number, i.e., larger heat transfer coefficients, that will result in short crystallization times. Overall, the results indicated operating at the lowest $T_{a}$ and the highest $V_{dg}$ to minimize the crystallization time. In other words, the results suggest operating at the extreme values of the process variables to realize the shortest crystallization times. However, this implies a high demand for energy. Therefore, an optimization with respect to the energy requirements could yield a more acceptable operating condition with a reasonable crystallization time.

## Figures and Tables

**Table 1:** Thermophysical properties of CB that have been used in the solidification model equations.

Parameter	Value
Specific heat capacity of liquid CB ($C_{p_{d}}$), [kJ/kgK]	2.2
Specific heat capacity of solid CB ($C_{p_{s}}$), [kJ/kgK]	1.25
Latent heat (*L*), [kJ/kg]	157
Density (ρ), [kg/m^3^]	894

**Table 2:** Data used in the model validation for CB droplet.

Parameter	Value
Droplet diameter, [mm]	2
Initial droplet temp. ($T_{ini}$), [K]	318
Start of solidification ($T_{f}$), [K]	291
End of solidification ($T_{s}$), [K]	273
Ambient air temp. ($T_{a}$), [K]	277
Ambient air pres. (*p*), [bar]	1.0
Inflow air vel. (*v*), [cm/s]	83

**Table 3:** Validation results for a single cocoa butter droplet.

Parameter	Simulation	Expt. [26]
Initial droplet cooling rate, [K/s]	6.3	7–9
Initial solidification cooling rate, [K/s]	0.5	0.5
Convective heat transfer coeff. (*h*), [W/m^2^K]	96.86 ^1^	110–135 ^2^
Nusselt number (Nu)	7.85 ^1^	9–11 ^3^

^1^ Average value computed over all the stages. ^2^ Estimated using Equation (12). ^3^ Estimated using Equation (13).

**Table 4:** Value of pre-constants, *A* and *B*, in Equations (14) and (15) for the various temperature-velocity combinations.

Ambient Temperature [K]	Inflow Velocity [cm/s]	*A*	*B*
270	100	0.38	0.86
270	20	0.5	1.25
220	100	0.12	0.15
220	20	0.21	0.23

## Data Availability

The datasets generated for this study are available on request to the corresponding author.
